# Pyruvate: Ferredoxin oxidoreductase is involved in IgA-related microbiota dysbiosis and intestinal inflammation

**DOI:** 10.3389/fimmu.2022.1040774

**Published:** 2022-12-07

**Authors:** Kairuo Wang, Yixuan Guo, Yuanyuan Liu, Xiao Cui, Xiang Gu, Lixiang Li, Yanqing Li, Ming Li

**Affiliations:** ^1^ Department of Gastroenterology, Qilu Hospital, Shandong University, Jinan, Shandong, China; ^2^ Department of Gastroenterology, Shanghai Tenth People's Hospital, School of Medicine, Tongji University, Shanghai, China; ^3^ Laboratory of Translational Gastroenterology, Qilu Hospital, Shandong University, Jinan, Shandong, China; ^4^ Robot Engineering Laboratory for Precise Diagnosis and Therapy of Gastrointestinal Tumor, Qilu Hospital, Shandong University, Jinan, Shandong, China; ^5^ Shandong Provincial Clinical Research Center for digestive disease, Qilu Hospital, Shandong University, Jinan, Shandong, China

**Keywords:** ulcerative colitis, *Faecalibacterium prausnitzii*, pyruvate: ferredoxin (flavodoxin) oxidoreductase, immunoglobin A, dysbiosis

## Abstract

**Introduction:**

Inflammatory bowel diseases (IBDs) are associated with both immune abnormalities and dysbiosis, characterized by a loss of Faecalibacterium prausnitzii (F. prausnitzii). However, the reason for F. prausnitzii deficiency remains unclear.

**Methods:**

16S rDNA seque­ncing and IgA enzyme-linked immunosorbent assay (ELISA) were applied to identify bacterial community and IgA changes in ulcerative colitis (UC) patients. Forced immunization with F. prausnitzii in rabbits was conducted. To screen for potential IgA-reactive proteins in F. prausnitzii lysates, we performed western blotting and mass spectrometry analyses. Pyruvate: ferredoxin oxidoreductase (PFOR) was cloned and purified, then the immunoreactivity of PFOR was verified in peripheral blood mononuclear cells (PBMCs) through PCR, ELISpot assay and single-cell sequencing (scRNA-seq). Finally, the UC fecal dysbiosis was re-analyzed in the context of the phylogenetic tree of PFOR.

**Results:**

F. prausnitzii was underrepresented in UC patients with elevated F. prausnitzii-reactive IgA in the fecal supernatant. Forced immunization with F. prausnitzii in rabbits led to high interferon-γ (IFN-γ) transcription in the colon, along with beta diversity disturbance and intestinal inflammation. PFOR was identified as an IgA-binding antigen of F. prausnitzii and the immunoreactivity was validated in PBMCs, which showed elevated expression of inflammatory cytokines. The scRNA-seq revealed enhanced signals in both T regulatory cells (Tregs) and monocytes after PFOR incubation. Furthermore, phylogenetic analysis revealed that PFOR was a common but conserved protein among the gut bacteria.

**Discussion:**

Our results collectively suggest that PFOR is a bioactive protein in the immune system and may contribute to host-microbial crosstalk. Conserved but bioactive microbial proteins, such as PFOR, warrant more attention in future host-microbial interaction studies.

## Introduction

Inflammatory bowel diseases (IBDs), including ulcerative colitis (UC) and Crohn’s disease, are immune-mediated, and several environmental factors are involved in their pathogenesis (1). Previous studies have documented that alterations in the gut microbiota contribute to the initiation and progression of UC as well as many other diseases. The fecal microbiota of UC patients differs significantly from that of healthy subjects, characterized by a predominant deficiency of commensal bacteria *Faecalibacterium prausnitzii* (*F. prausnitzii*), which is one of the most abundant bacteria in the human gut microbiota, accounting for 1.4 to 5.9% of the total bacterial population (2, 3). Defective gut colonization by *F. prausnitzii* is associated with less remission and more relapse in UC patients (4). *F. prausnitzii* has been shown to protect against UC *via* the production of butyrate and anti-inflammatory proteins (4–6). However, the precise mechanism underlying *F. prausnitzii* deficiency in UC remains unclear.

Secretory immunoglobulin A (IgA) is the predominant antibody isotype produced at mucosal surfaces and is essential for maintaining gut homeostasis by protecting against invading pathogens while tolerating commensal bacteria, which mediates immune-microbiota crosstalk (7, 8). IgA has been used to identify the outer membrane proteins of Shiga toxin-producing *Escherichia coli* expressed during human infection *via* immunoproteomic analysis (9). In our previous study, fecal IgA in irritable bowel syndrome (IBS) patients was used to identify the key antigen of *Fusobacterium nucleatum* (*F. nucleatum*), which exacerbates visceral hypersensitivity in a colonization-independent manner (10). Thus, IgA is valuable for identifying disease-associated bacteria and discovering crucial antigens (11, 12). In patients with UC, the proportion of intestinal bacteria coated with IgA is significantly increased (13). *F. prausnitzii* is one of the bacterial species recognizable by host IgA (14). However, the key proteins in *F. prausnitzii* that are reactive to IgA remain unclear.

In this study, we present evidence for dysbiosis in the gut microbiota of UC patients with a reduced abundance of *F. prausnitzii* along with an increased IgA response. Subcutaneous immunization with *F. prausnitzii* lysate causes intestinal inflammation and microbiota changes in rabbits. Furthermore, we identify pyruvate: ferredoxin (flavodoxin) oxidoreductase (PFOR) as the principal IgA-binding antigen of *F. prausnitzii*, which is highly potent in activating peripheral blood mononuclear cells (PBMCs). Single-cell sequencing (scRNA-seq) of PBMCs revealed enhanced signals in both T regulatory cells (Tregs) and monocytes after PFOR incubation. In addition, PFOR is a ubiquitous but conserved protein among bacteria. Altogether, our results suggest that the PFOR protein plays an important role in host-microbial crosstalk. Other conserved microbial proteins may act in the same way, and these bioactive proteins deserve more attention in future microbiota analysis as potential intervention targets.

## Materials and methods

### Patients

The human subjects included 36 UC patients and 28 healthy controls. All patients were from the Department of Gastroenterology, Qilu Hospital, Shandong University (Jinan, Shandong, China). The baseline characteristics of all participants are described in **Supplementary Table 1**. The diagnosis of UC was based on clinical, laboratory, endoscopic examinations, and histological findings. Disease severity was assessed according to Mayo scores for patients with UC. The exclusion criteria included pregnancy; lactation; previous surgeries within half a year; vaccination within three months; and taking antibiotics, probiotics, and laxatives within a month. Written informed consent was obtained from all participants prior to their enrollment in the study. Studies involving human participants were reviewed and approved by the ethics committee of the Qilu Hospital, Shandong University (KYLL-2017(KS)-006).

### Animals

Male New Zealand white rabbits were bred and maintained under specific-pathogen-free conditions at the Qilu Hospital of Shandong University (Jinan, Shandong, China). Animals were randomly selected from all experimental groups. All animals were kept in separate cages and fed a standard laboratory rabbit maintenance diet (Cat# 101003, Xietong Bio, Nanjing, China) under standardized conditions with regulated daylight, humidity, and temperature. All procedures in this study were performed in accordance with the guidelines of the Chinese Institutional Animal Care Committee. The animal studies were reviewed and approved by the ethics committees of Qilu Hospital, Shandong University (DWLL-2018-017).

### Bacterial strains and culture

*Faecalibacterium prausnitzii* strain A2-165 (DSMZ 17677) was grown overnight at 37°C in YBHI broth (brain-heart infusion medium supplemented with 0.5% yeast extract) supplemented with 1 g/L cellobiose, 1 g/L maltose, and 0.5 g/L L-cysteine (all from Sigma-Aldrich) in an anaerobic chamber using a mixture of hydrogen (H_2_) and nitrogen (N_2_) (5/95%). *Escherichia coli* (*E. coli*) DH5α and *E. coli* Rosetta2 (DE3) were purchased from Beyotime Biotechnology and cultured aerobically at 37°C in Terrific Broth (TB).

### 16S rRNA sequencing

Human fecal microbiota samples were processed at Majorbio (Shanghai, China). A FastDNA SPIN kit (MP Biomedicals, California, USA) was used to extract stool DNA. Polymerase chain reaction (PCR) (ABI GeneAmp 9700, ABI, USA) was used to amplify the V3-V4 region of the bacterial 16S rRNA gene with barcode-indexed primers (338F: 5’-ACT CCT ACG GGA GGC AGC AG-3’, 806R: 5’-ACT CCT ACG GGA GGC AGC AG-3’) using TransStartFastPfu DNA Polymerase (TransGen, Beijing, China). The amplicons were then purified by gel extraction using the AxyPrep DNA Gel Extraction Kit (Axygen, California, USA) and quantified using QuantiFluor-ST (Promega, USA). The purified amplicons were pooled in equimolar concentrations, and paired-end sequencing was performed using an Illumina MiSeq PE300 system (Illumina, San Diego, USA).

The 16S rRNA gene pyrosequencing of the rabbit microbiota was performed using Novogene (Tianjin, China). Total metagenomic DNA from the samples was extracted using the CTAB/sodium dodecyl-sulfate (SDS) method. DNA concentration and purity were monitored using 1% agarose gels. According to the concentration, DNA was diluted to 1 ng/μL using sterile water. The V4 region of the 16S rRNA gene was amplified using specific primers with the barcode (515F: 5’-GTG CCA GCM GCC GCG GTA A-3’, 806R: 5’-GGA CTA CHV GGG TWT CTA AT-3’). All PCR reactions were carried out in 30 μL reactions with 15 μL of Phusion^®^ High-Fidelity PCR Master Mix (New England Biolabs), 0.2 μM of forward and reverse primers, and about 10 ng of template DNA. Thermal cycling consisted of an initial denaturation at 98°C for 1 min, followed by 30 cycles of denaturation at 98°C for 10 s, annealing at 50°C for 30 s, and elongation at 72°C for 30 s, with a final step of 72°C for 5 min. The PCR products were purified using the GeneJETTM Gel Extraction Kit (Thermo Scientific, Shanghai, China). Finally, the library was sequenced using the an Ion S5 XL platform.

### Sequence processing and microbiota quantification

Quality filtering of the raw reads was performed under specific filtering conditions to obtain high-quality clean reads, according to Cutadapt (V1.9.1). Raw reads were filtered using the following four criteria: 1) low-quality bases in the tail of raw reads were cut. If the average base quality in a sliding window of 50 bp was lower than 20, the bases were cut after the beginning of the window. If the remaining reads were shorter than 50 bp, the entire read was discarded. 2) The paired reads were merged, and the minimal overlap length was 10 bp. 3) The maximum mismatch ratio in the overlapping region was 0.2. 4) Merged reads were assigned to samples according to the barcodes at both ends. The maximum mismatch of the barcode was 0, and the maximum mismatch of the primers was 2.

After clean reads were obtained, sequence analysis was performed using USEARCH v11 according to the pipeline example (https://drive5.com/usearch/manual/ex_miseq.html). Briefly, the valid sequences were de-replicated by the fastx_uniques algorithm of usearch. The unique reads were denoised, and chimeras were removed using the unoise3 command to produce zero-radius operational taxonomy units (ZOTUs). Then, the representative sequence of each ZOTU was aligned to the RDP (version 18) using the sintax algorithm with a sintax_cutoff of 0.8. The ZOTU abundance was merged to genus and phylum levels using the sintax_summary algorithm with parameters -rank g and -rank p, respectively. Alpha and beta diversities were calculated using the usearch -alpha_div, -cluster_agg, and -beta_div algorithms. non-metric multidimensional scaling (NMDS) with adonis analysis was performed with the metaMDS, adonis, or anoism function of the vegan package v2.5 in R. LEfSe (linear discriminant analysis [LDA] coupled witheffect size measurements) analysis was conducted to calculate the biomarkers between the groups. Specifically, the starting formatted data file for LEfSe analysis was made from the usearch produced phylum, class, order, family, and genus level summary files using hand crafted code in R. Then by running the script LEfSe script from github (https://github.com/SegataLab/lefse/tree/master/lefse) in python3, the LDA score of biomarkers was calculated and plotted. A stricter all-against-all strategy was adopted for the multi-class comparison. Biomarkers with an LDA score > 2.0 were filtered and plotted.

The functional capacity of the human microbiota was predicted using the Tax4Fun algorithm (15). Briefly, representative sequences were aligned with SILVA v123 reference data. Then, the biome data were imported to R and calculated using the Tax4Fun function with parameter fctProfiling=TRUE. The abundance of pyruvate ferredoxin oxidoreductase (PFOR) subunit alpha to gamma (K00169 to K00172) was summed as the abundance of PFOR.

### Fecal supernatants preparation

Fresh fecal pellets from humans and rabbits were homogenized in phosphate-buffered saline (PBS) supplemented with 0.04 mg/mL soybean trypsin inhibitor, 20 mM ethylenediaminetetraacetic acid (EDTA), and 2 mM phenylmethanesulfonyl fluoride (PMSF) and centrifuged to remove bacteria at 12000 g for 15 min. The lysates were sterilized by filtration through 0.2-μm syringe filters.

### ELISA for fecal IgA

Enzyme-linked immunosorbent assays (ELISAs) for the quantitative detection of human fecal IgA and rabbit fecal IgA were conducted using ELISA kits (Abcam) according to the manufacturer’s instructions.

To assess specific IgA responses, 96-well plates (Nunc Maxisorp) were coated with 50 μg/μL collagen I (rat tails; Gibco) in 20 mM acetic acid overnight on a shaker at 4°C. For *F. prausnitzii* IgA ELISA analysis, the plates were washed twice with PBS before adding 10^9^ bacteria/mL in PBS (bacteria were cultured overnight prior to the experiment). The plates were then incubated overnight at 37°C under anaerobic conditions. For the PFOR IgA ELISA, plates were coated with 5 μg/mL PFOR overnight at 4°C. After incubation, the plates were rinsed thrice with PBS and fixed in 4% paraformaldehyde. The fixative was removed, and the plates were rinsed three times with PBS before blocking with 5% milk in PBST buffer (PBS with 0.05% Tween 20) for 1 h at 37°C on a shaker. After dilution in 2% milk, fecal lysates were added to the plates and incubated for 2 h at room temperature on a shaker. After washing, adherent antibodies were detected using horseradish peroxidase (HRP)-goat anti-human IgA or HRP-goat anti-rabbit IgA antibody (Abcam) at 1:1000 in 2% milk for 1 h at 37°C. The plates were rinsed three times in PBS and developed with TMB substrate. The reaction was stopped by adding 1 N H_3_PO_4_, and the absorbance was measured using an ELISA reader at 450 nm.

### Immunization and challenge

In the *F. prausnitzii* immunization experiment, the rabbits were subcutaneously injected with three types of agents: 1) sterile PBS solution for the PBS group (n=5); 2) 2 mg/mL QuilA solution dissolved in PBS for the QuilA group (n=5); and 3) 10^9^ centrifugation-concentrated *F. prausnitzii* solution dissolved in 1 mL of 2 mg/mL QuilA solution for the QuilA + FP group (n=6). After body weight and fecal sample collection on day 0, each rabbit was subcutaneously injected with different agents on the cervical back skin (0.3 mL/kg). Body weight was measured daily, and fecal samples were collected on days 0, 1, 2,4 and 8. The rabbits were sacrificed on day 10, and the ileum, cecum, and colon tissues were collected. The tissue specimens used for hematoxylin and eosin (H&E) staining were fixed in 4% paraformaldehyde overnight and then embedded in paraffin. The tissues used for qPCR analysis were rapidly frozen in liquid nitrogen and stored at -80°C.

### Histological assessment

Tissues were fixed overnight at 4°C in 4% paraformaldehyde, embedded in paraffin, sectioned, and stained with H&E. Histological analyses of morphological features and inflammation were performed by a board-certified pathologist according to a modified criterion described in a previous publication (16). The scoring system is shown in **Supplementary Table 2**.

### Total RNA extraction and real-time quantitative PCR analysis

Total RNA was extracted from tissue specimens using an RNAprep Pure Tissue Kit (TIANGEN), according to the manufacturer’s protocol. Total RNA was extracted from cells using the RNAprep Pure Cell/Bacteria Kit (TIANGEN). cDNA was synthesized using a ReverTra Ace qPCR RT Kit (Toyobo, Osaka, Japan). All reactions were run in triplicate on an Applied Biosystems StepOne Real-Time PCR System (Thermo Fisher Scientific, Waltham, MA, USA). The sequences of the PCR primers are listed in **Supplementary Table 3**. qPCR data were analyzed using the 2^–ΔΔCT^ method with GAPDH as the housekeeping gene. The thermocycler program was as follows: 95°C for 1 min, followed by 40 cycles at 95°C for 15 s, 60°C for 15 s, and 72°C for 45 s.

### Western blot

Late-log cultures of *F. prausnitzii* were pelleted at 8000 g for 5 min and resuspended at a concentration of 10^10^ bacteria/mL in PBS. The precipitate was mixed at a 1:1 ratio with 2× protein loading buffer and boiled for 10 min to lyse the bacteria. Twenty microliters of lysate were loaded per well in Tris-glycine gels and run in Tris-glycine buffer with 0.1% SDS. The gels were transferred to a polyvinylidene difluoride (PVDF) membrane on ice for 2 h in the same buffer supplemented with 20% methanol. The PVDF membrane was blocked for 5 h in PBST (PBS with 0.1% Tween 20) with 5% non-fat milk, washed with PBST, and then coated with the fecal supernatant (total protein concentration of 0.1 μg/mL) overnight at 4°C. After washing, the membrane was stained with HRP-labeled IgA (Abcam) diluted 1:1000 in PBST with 5% powdered milk for 1 h, washed five times again, and developed using an enhanced chemiluminescence kit (Merck Millipore).

### Identification of proteins from gel sample

For antigen identification, the proteins of interest were excised from the gels after Coomassie brilliant blue staining and digested with trypsin (Promega, USA) in digestion buffer (ammonium bicarbonate 100 mM, pH 8.5). The peptides from the digestion were extracted with acetonitrile and dried completely. The dried samples were then re-dissolved. The sample was then digested using sequencing-grade modified trypsin. The dissolved peptide sample was analyzed using a NanoLC-ESI-MS/MS system. Mass spectrometric data were used to search against the UniProt protein database using ProtTech’s ProtQuest software suite.

### Recombinant protein expression

PFOR coding sequences were amplified from *F. prausnitzii* DNA using the following primer pair: 5’-TTT ATT TTC AAG GTA AGC TTA TGC CTA GAG CAA AGC AAA CCA TG-3’ and 5’-TGG TGG TGG TGG TGC TCG AGT TAC TTG TAC AGC TCG ACC AGA CG-3’. Additionally, fragments of PFOR were amplified. The primer pairs were as follows: 5’-TTT ATT TTC AAG GTA AGC TTA TGC CTA GAG CAA AGC AAA C-3’ and 5’-TGG TGG TGG TGG TGC TCG AGT TAC TCA CGC AGG GTA GCA GCA A-3’ for fragment 1 (0–350 bp); 5’-TTT ATT TTC AAG GTA AGC TTA CCA AGG AGC CCG GCT CCC T-3’ and 5’-TGG TGG TGG TGG TGC TCG AGT TAA GGA ACC ATA GCA ATT GCC T-3’ for fragment 2 (330–770 bp); 5’-TTT ATT TTC AAG GTA AGC TTG ACT GCA TGG GCT GCG GCG A-3’ and 5’-TGG TGG TGG TGG TGC TCG AGT TAC TTG TAC AGC TCG ACC AGA C-3’ for fragment 3 (750 bp-C-terminal). HindIII and XhoI restriction sites were added at the 5’ and 3’ ends of all the fragments, respectively.

The PCR products were double-digested and found to be recombinant with the pET-N-His expression vector using a 2× seamless cloning kit (Abiotech, Jinan, China). The pET-N-His plasmid was created based on the pET vector, and an N-terminal His-tag sequence was carried out. The vector construct was transformed into *E. coli* DH5α cells cultured in TB medium containing 100 μg/mL ampicillin. The amplified pET-N-His construct was purified from cultured cells and transformed into *E. coli* Rosetta2 (DE3) cells cultured in TB containing 100 μg/mL ampicillin. The cells were cultured at 37°C until reaching an OD 600 of 0.6, after which 0.1 mM isopropyl-1-thio-b-D-galactopyranoside (IPTG) was added, and the cells were cultured at 37°C for 4 h to induce PFOR protein expression. The collected bacterial solution was centrifuged to obtain a precipitate, which was ultrasonically lysed in lysis buffer (10 mM Tris-HCl, pH 8.0, 500 mM NaCl). After centrifugation at 4°C, the supernatant was purified on a nickel column (Abiotech, Jinan, China), washed with washing solution (10 mM Tris-HCl, pH 8.0, 500 mM NaCl, and 20 mM imidazole) to remove heterologous proteins, and eluted with elution buffer (10 mM Tris-HCl, pH 8.0, 500 mM NaCl, and 500 mM imidazole). Eluted PFOR proteins were confirmed by SDS-PAGE and Coomassie blue staining. The eluted PFOR proteins were collected, dialyzed against a dialysis solution (10 mM Tris-HCl, pH 8.0, 500 mM NaCl, and 10% glycerol), and stored at -20°C.

### ELISpot assay

PBMCs were isolated from healthy blood donors using Ficoll-Paque density gradient centrifugation. The cells were washed and resuspended in RPMI 1640 medium (Gibco) supplemented with 10% fetal bovine serum (FBS), penicillin (100 U/mL), and streptomycin (100 mg/mL). IFN-γ ELISpot assays were performed according to standard protocols (Mabtech). Sterile pre-coated 96-well ELISpot plates were washed four times with sterile PBS (200 μL/well) and blocked with 200 µL of RPMI 1640 supplemented with penicillin, streptomycin, and 10% FBS for 1 h at RT. PBMCs were counted and adjusted to a density of 5×10^6^ cells/ml. Fifty microliters of the stimulus (medium or 25 ng/μL PFOR) and 50 µL of PBMCs were added to the plate. The plate was incubated overnight at 37°C in a 5% CO_2_ incubator and then washed five times before adding 100 µL of the detection antibody at 1 μg/mL in PBS containing 0.5% FBS. The plate was incubated for 2 h at °C and washed. Streptavidin-horseradish peroxidase (HRP) (100 μL streptavidin-HRP (1:1000)) was added and incubated for 1 h. Finally, 100 μL of TMB substrate solution was used for 20 min, and the reaction was stopped by thoroughly washing the plate in deionized water. The plate was dried overnight, and spot-forming cells were counted using an Immuno-Spot reader.

### Single-cell RNA sequencing and data analysis

In this study, scRNA-seq of 10× Genomics based on droplet capture technology was performed by Novogene (Tianjin, China), according to the manufacturer’s standard protocol (17). Briefly, the harvested single cells and gel beads with barcoded sequences and oil droplets were placed together in the machine. These gel beads precisely captured the target cells. Then, the cells were lysed and released a large quantity of RNA and barcoded sequences, which underwent reverse transcription to produce cDNA templates for sequencing. Bulk PCR amplification was then performed to establish a library for 3’-end sequencing.

The comparison software Cellranger (version 5.0.0) was used to align the reads to the Genome Reference Consortium Human Build 38 (GRCh38). The R package Seurat was used to remove batch effects and perform normalization, canonical correlation analysis (CCA), clustering analysis, uniform manifold approximation and projection (UMAP) visualization, and differential gene analysis. The scRNA-seq data were visualized in violin plots, UMAP plots, dot plots, and heat maps using the FeaturePlot function in Seurat. The cell types were automatically annotated *via* scCATCH and manually corrected according to their marker genes. The transcription of key genes involved in immune and inflammatory processes was extensively analyzed, and potentially meaningful results are shown in a violin plot.

### Phylogenetic analysis

The bacterial PFOR protein sequences were retrieved by searching “Pyruvate: ferredoxin oxidoreductase family” in the UniProtKB database (https://www.uniprot.org/). Hits were downloaded and manually reviewed for protein and strain names. The genus names of the PFOR-containing organisms were summarized and intersected with the genus names present in the human fecal microbiome, thus obtaining the human microbiome-related PFOR proteins (h-PFOR). The phylogenetic tree of h-PFOR was visualized using the ggtree package in R (18). All h-PFOR sequences were blasted against the PFOR gene of *F. prausnitzii* A2-165 using blastp (v 2.13.0). Protein identity and coverage of the top 500 hits were summarized and plotted in R. The genera containing a PFOR gene with >80% identity were selected and compared in our IBD control cohort.

### Statistical analysis

Data are reported as the mean ± SEM. Statistical analyses were performed using IBM SPSS Statistics (version 26.0) and R software (version 4.2.1). The receiver operator characteristic (ROC) curve and area under the curve (AUC) were modeled using R software with the ROCR package. Differences between two groups were evaluated using Student’s *t*-test, Welch’s *t*-test (parametric), or Wilcoxon test (non-parametric). For comparison of more than three groups, a one-way ANOVA (parametric) or Kruskal-Wallis test (non-parametric) was used, followed by Dunn’s test for non-parametric samples as a *post hoc* test. For the batch comparison of taxonomic abundances, the Kruskal-Wallis test was adopted, and the p values were adjusted by the “fdr” method in p.adjust function in R. Differences with p < 0.05 were considered significant in general comparison, and differences with p < 0.025 were considered significant in Dunn’s *post hoc* pairwise comparison.

## Results

### A decrease of *Faecalibacterium* with increased IgA responses in UC patients

**Figure 1:**
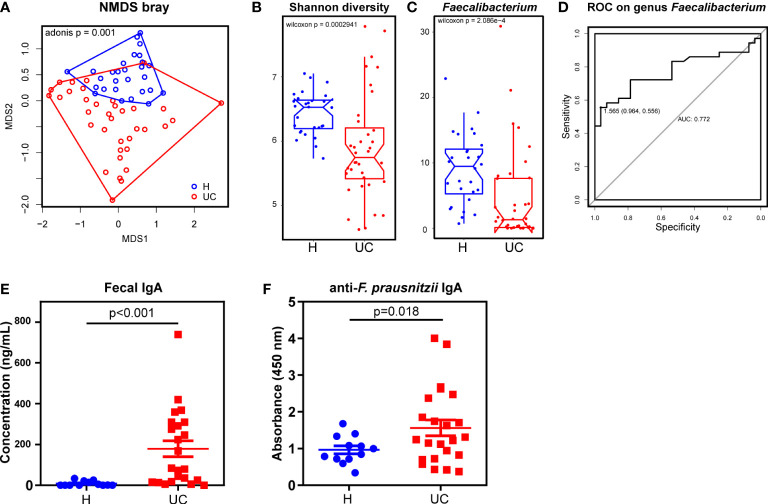
A decrease of *Faecalibacterium* with increased IgA responses in UC patients. **(A)** NMDS analysis and the Adonis p value based on Bray distance. **(B)** The Shannon diversity. **(C)** Genus *Faecalibacterium* comparison between UC and the healthy control. **(D)** The ROC curve of diagnosing UC by genus *Faecalibacterium*. **(E)** ELISA test of total IgA in fecal supernatants (Wilcoxon p < 0.001). **(F)** ELISA test of *F. prausnitzii*-reactive IgA in fecal supernatants (Wilcoxon p=0.018). Data are shown as means ± SEM.

We began by assessing dysbiosis in patients with UC. While patients with UC had a significantly lower body mass index than healthy patients, these recipients were comparable in sex and age. The basic characteristics are summarized in **Supplementary Table 1**. Using NMDS analysis based on Bray distance reflecting beta diversity, we found that the UC microbiota were more dispersed than the healthy microbiota (**Figure 1A**). Consistent with previous studies, the alpha diversity (Shannon diversity) was significantly lower in UC patients than in healthy individuals (**Figure 1B**). Next, we tested the taxonomic differences between UC and healthy fecal microbiota. The abundance of *Faecalibacterium* was significantly lower in patients with UC than in healthy individuals (**Figure 1C**). The abundance of the two groups at the genus level is shown in **Supplementary Data 1**. We then tested the discrimination robustness between the two groups based on the *Faecalibacerium* abundance receiver operating characteristic (ROC) curve (**Figure 1D**). When diagnosing UC by fecal *Faecalibacerium* abundance, the optimal cut-off value was 1.565%, with a sensitivity of 55.6%, specificity of 96.4%, and AUC of 0.772. With high specificity, this model provided a high positive predictive value and was an ideal property of a “rule-in” test. However, the sensitivity was low, indicating poor ability to identify patients with UC. Therefore, the *F. prausnitzii* abundance could be used as a candidate value in combination with other clinical values to construct a diagnostic tool. To test fecal IgA levels, we performed an ELISA, which showed that fecal IgA concentrations were substantially higher in UC patients than in controls (**Figure 1E**). We next tested *F. prausnitzii-*specific IgA responses in fecal supernatants using ELISA and found that UC patients had a significantly higher IgA response to *F. prausnitzii* lysates (**Figure 1F**).

### *F. prausnitzii* immunization caused elevated IgA levels and beta diversity disturbance

**Figure 2:**
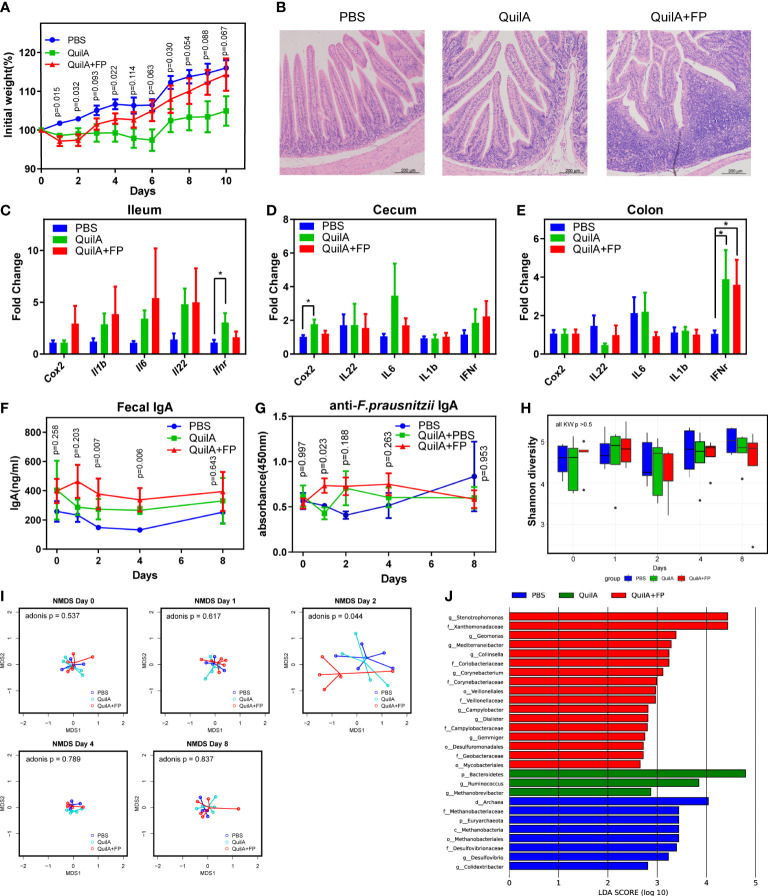
F*. prausnitzii* immunization caused elevated IgA levels and beta diversity disturbance. **(A)** Body weight growth rate. **(B)** H&E staining of ileum section. **(C–E)** The expression of inflammation-related genes of the ileum, cecum, and colon evaluated by RT-PCR. **(F)** IgA level in fecal supernatants evaluated by ELISA test. **(G)** Anti-*F. prausnitzii* IgA level in fecal supernatants evaluated by ELISA test. **(H)** Comparison of Shannon diversity. **(I)** NMDS test based on Euclidean distance with p values of Adonis test. **(J)** Histogram of the linear discriminant analysis (LDA) scores among PBS, QuilA, and QuilA + FP groups. Data are shown as means ± SEM. * p < 0.05.

We wondered whether the reduced colonization of *F. prausnitzii* was a result of aberrant IgA. We found that *F. prausnitzii* could not persistently colonize the mouse gut (data not shown) but was a common OTU in the fecal microbiota of most rabbits. Therefore, rabbits were chosen for animal experiments (19). In this study, QuilA adjuvant was used to immunize rabbits with *F. prausnitzii*. QuilA is capable of inducing both cell- and antibody-mediated immune responses to a wide range of antigens, including mucosal antigens (20). All rabbits survived until the 10th day after immunization, except one in the QuilA + FP group, which died on the 6th day after immunization. We found that QuilA and QuilA + FP delayed the body weight increase in the initial 2 days after immunization (**Figure 2A**). In the later days, rabbits recovered weight growth by an insignificantly larger extent in the QuilA + FP group than in the QuilA group (**Figure 2A**). However, body weight does not necessarily reflect histological inflammation. For example, there may be a slight increase in the body weight of mice with dextran sulfate sodium (DSS)-induced colitis in the initial days (21). Thus, histological analysis and qPCR were conducted to further evaluate the inflammatory state. As expected, histological analysis revealed that the QuilA + FP group had the largest ileum and total intestinal scores (**Figure 2B** and **Supplementary Figure 1A**). The PCR results showed that QuilA immunization increased the expression of IFN-γ in the ileum and Cox-2 in the cecum (**Figures 2C, D**). In addition, the QuilA and QuilA + FP groups showed overexpressed IFN-γ in the colon compared to that in the PBS group (**Figure 2E**).

We then examined whether our forced immunization model elicited fecal IgA levels. Using ELISA, we found that the QuilA + FP group had the highest total IgA at the 2nd and 4th day after immunization (**Figure 2F**). By the 8th day, the difference in total IgA in fecal supernatants was again insignificant. By measuring *F. prausnitzii*-reactive IgA levels, we found a temporally but significantly higher level on the 1st day in the QuilA + FP group (**Figure 2G**). These data indicate that forced immunity to *F. prausnitzii* induced non-lasting anti-*F. prausnitzii* IgA the next day, partially replicating abnormal immunity in patients with UC.

We then evaluated whether this model replicated any dysbiotic features in patients with UC. Shannon diversity and *Faecalibacterium* genus abundance were not different among the three groups throughout this experiment (**Figure 2H** and **Supplementary Figure 1B**). By Bray distance-based NMDS analysis, we found that the fecal microbiota of the QuilA + FP group deviated from the other groups at day 2 but not at days 0, 1, 4, or 8 (**Figure 2I**). The adonis test confirmed dysbiosis in the QuilA + FP group on day 2 (p = 0.044). We then performed LEfSe analysis of the microbiota on day 2 and found that the QuilA + FP group was characterized by overrepresentation of *Stenotrophomonas*, *Geomonas*, *Mediterraneibacter*, *Collinsella*, *Corynebacterium*, *Campylobacter*, *Dialister*, and *Gemmiger* at the genus level (**Figure 2J**, all LDA scores >2.5). These data showed that forced immunity to *F. prausnitzii* caused elevated IgA levels and temporal fecal microbiota disturbance in the feces.

### PFOR is a major antigen of *F. prausnitzii* reacted with fecal IgA

**Figure 3:**
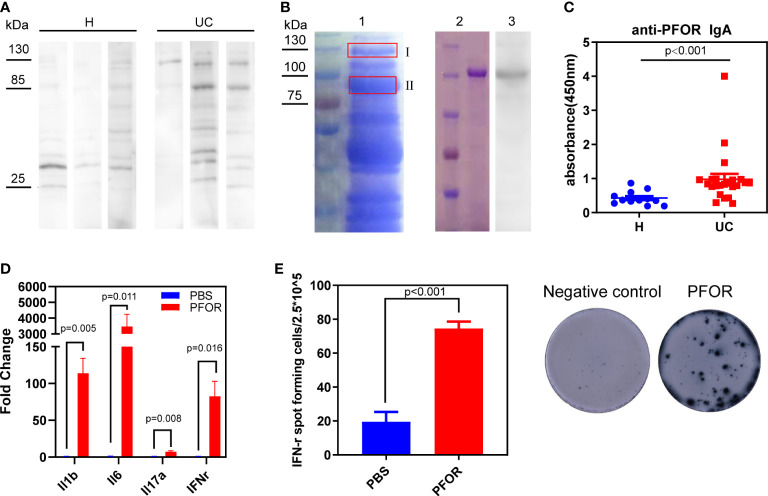
PFOR is a potential antigen of *F. prausnitzii* reacted with fecal IgA. **(A)** Representative images of western blot using *F. prausnitzii* lysates as substrates, UC or healthy fecal supernatants as primary antibody, and anti-IgA antibody as secondary antibody. **(B)** Lane 1 was the electrophoresis of *F. prausnitzii* lysates stained by Coomassie brilliant R250. Two gel bands were isolated, and the corresponding mass spectrum results were shown in **Supplementary Data 2** and **Supplementary Table 4**. Lane 2 was the purified PFOR from transgenic *Escherichia coli* Rosetta (DE3), stained by Coomassie brilliant R250. Lane 3 was the western blot using PFOR as substrates and UC fecal supernatants as the primary antibody. **(C)** ELISA test of anti-PFOR IgA in fecal supernatants (Wilcoxon p < 0.001). **(D)** Evaluation of inflammation-related genes, including IL-1β, IL-6, IL-17α, and IFN-γ by RT-PCR. **(E)** ELISpot assay detecting IFN-γ expression in PBMCs. Data are shown as means ± SEM.

To test whether some *F. prausnitzii* antigens were specifically recognized by fecal IgA, we performed western blotting. At molecular masses of 85 and 130 kDa, strong reactive bands were detected in the UC fecal supernatants but less so in the healthy supernatants (**Figure 3A**). Furthermore, the correlations between *Faecalibacterium* abundance and the intensity of bands at 85 and 130 kDa were negative, with no statistical significance (**Supplementary Figure 2A, B**). Thus, we suspected that some *F. prausnitzii* antigens of 85 and 130 kDa were targets that reacted with fecal IgA. To further identify the *F. prausnitzii* proteins with these two molecular masses, we isolated the two gel bands for mass spectrometry (**Figure 3B**, lane 1). The PFOR protein was most frequently detected in the 130 kDa band and was also present in the 85 kDa band (**Supplementary Table 4**, **Supplementary Data 2**). We also analyzed the genome of *F. prausnitzii* in the National Center for Biotechnology Information (NCBI) database (assembly ASM16201v1). PFOR contains 1181 amino acids with an estimated molecular weight of 129.9 kDa. Previous reports have documented that the PFOR of *Helicobacter pylori* is capable of inducing an early immune response in infants (22). Thus, the PFOR of *F. prausnitzii* is suspected to be one of the major antigens recognized by fecal IgA.

To test this hypothesis, we cloned and purified PFOR from *F. prausnitzii*. Purified PFOR had a molecular weight of 130 kDa (**Figure 3B**, lane 2). It was reactive to the UC fecal supernatant IgA but less reactive to the healthy supernatants (**Figure 3B**, lane 3 and **Supplementary Figure 3**). We next found significantly elevated anti-PFOR IgA levels in UC fecal supernatants compared to those in healthy controls (**Figure 3C**). We then analyzed fecal microbiota data and predicted the metagenomic abundance of the PFOR gene using the Tax4Fun algorithm (15). We found that the abundance of PFOR was significantly lower in UC microbiota than in healthy microbiota (**Supplementary Figure 4**). These data collectively suggest that the PFOR protein has the potential to react with IgA in the feces of patients with UC. We then investigated the effects of PFOR on immune cells. PBMCs from five healthy donors were cultured *in vitro* and stimulated with purified PFOR protein. Using qPCR, we found that PFOR significantly increased the transcription of inflammatory cytokines, including IL-1β, IL-6, IL-17α, and IFN-γ (**Figure 3D**). The ELISPOT assay confirmed the IFN-γ-stimulating effect of PFOR (**Figure 3E**). Meanwhile, we cloned and purified three fragments of PFOR and evaluated IFN-γ expression using the ELISpot assay, which showed that each fragment had no obvious immunostimulatory effects (**Supplementary Figure 5A, B**). These data confirmed that the intact PFOR of *F. prausnitzii* is capable of activating PBMCs.

### The effects of PFOR on PBMCs revealed by single-cell sequencing

**Figure 4:**
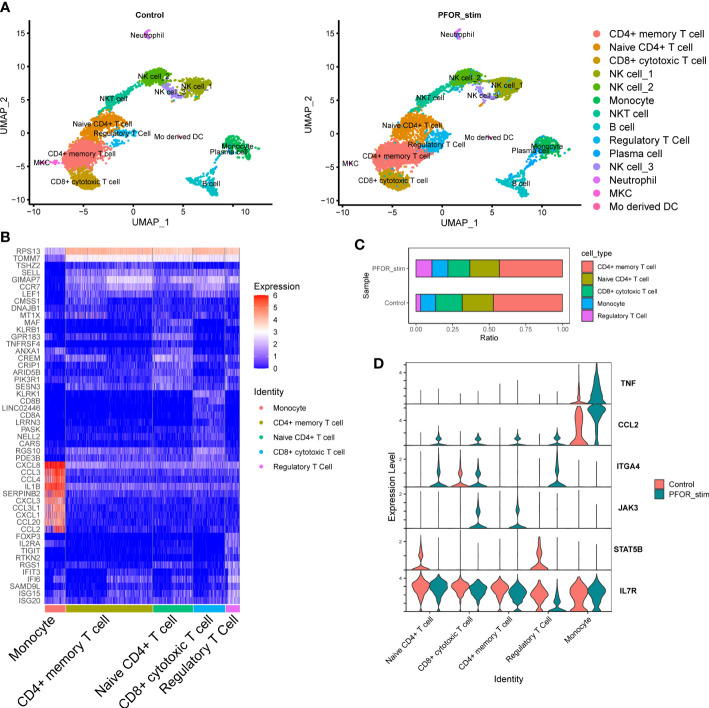
The effects of PFOR on PBMCs revealed by single-cell sequencing. **(A)** The UMAP reduction of control and PFOR-stimulated PBMCs. Each dot represents a sequenced cell, and it is colored according to the cell type (common legend right-side). **(B)** Heatmap plot of the differentially expressed genes in the five major clusters. **(C)** Bar plot showing the percentage of the five major cell clusters. **(D)** Violin plot of key inflammation and immune genes. X axis represents the cell clusters and Y axis the transcription level.

To fully reveal the effects of PFOR on immune cells, we used single-cell RNA sequencing (scRNA-seq) of two PBMC samples from one healthy donor treated with the PFOR protein or fragment 2, which is inactive (as a control). After quality control and filtering by multiple criteria, transcriptomes of 9235 and 6771 cells from the PFOR- and control-stimulated PBMCs were acquired, with a median of 1446 and 1291 genes per cell detected, respectively. The two single-cell datasets were merged, and the UMAP algorithm was used to cluster cells with similar expression patterns by dimension reduction (**Figure 4A**, **Supplementary Figure 6**). All cells from the two samples were homogeneous after integration and were classified into 14 clusters (**Figure 4A**, **Supplementary Figure 6**). No specific unique cell clusters were found in either sample. The 14 clusters were annotated based on the expression of gene markers using scCATCH and manual correction (**Figure 4B**). Five major clusters, monocytes, CD4^+^ memory T cells, naïve CD4^+^ T cells, CD8^+^ cytotoxic T cells, and regulatory T cells (Tregs) were selected for the following analysis.

By comparing the composition of cell subpopulations, we noticed an obviously increased proportion of Tregs in the PFOR-stimulated sample compared with the control sample (10.87% vs. 3.19%, **Figure 4C**). This trend was significant in the per-cell Chi-squared test (df = 4, p < 2.2×10^-6^). Enriched in the intestinal lamina propria, Treg cells promote intestinal B cell IgA production through secretion of TGF-β (23). Depletion of Treg cells results in a decrease in lamina propria IgA^+^ B cells as well as total commensal bacterial antigen-specific secretory IgA production (24, 25). Therefore, PFOR-stimulated Treg cell development may play an essential role in the production of intestinal IgA and the maintenance of intestinal immune homeostasis. We then analyzed the transcription of well-known genes in five clusters involved in the inflammatory process. We observed a trend for increased TNF gene transcription in PFOR-stimulated monocytes (**Figure 4D**). CCL2 transcription tended to increase in T cells and monocytes. The integrin α4 gene, ITGA4, was increased, while that of STAT5B was decreased in naïve CD4^+^ T cells and Tregs. PFOR was capable of activating JAK3 in CD8^+^ cytotoxic and CD4^+^ memory T cells. In addition, IL7R tended to decrease Tregs. Taken together, stimulation with PFOR induced an immune response in multiple cell types, as indicated by the changes in chemotaxis and homing genes.

### PFOR is conserved and ubiquitous in the gut microbiome

**Figure 5:**
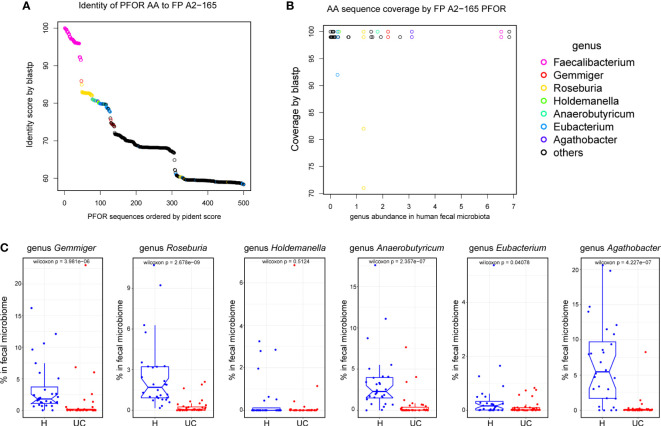
PFOR is conserved and ubiquitous among the gut microbiota. **(A)** Identity of PFOR AA to *F. prausnitzii* A2-165. **(B)** AA sequence coverage by *F. prausnitzii* A2-165 PFOR. **(C)** The abundances of PFOR-containing genus in human recipients. Data are shown as means ± SEM.

Because the PFOR of *F. prausnitzii* is bioactive, we wondered if other members of the microbial community have PFOR genes. We searched for PFOR proteins by name in the Uniport database. After manual review, we got 35, 230 PFOR protein sequences with organisms belonging to 2182 genera. We then reanalyzed the fecal microbiota of the IBD and control cohorts. We found 156 genera in the human microbiota, 117 of which were shared with PFOR^+^ genera (**Supplementary Figure 7**). We filtered the PFOR sequences in these genera and obtained 4936 that were deemed human-related (**Supplementary Data 3**). The phylogenetic tree of all human-related PFOR was plotted with the PFOR sequences of *F. prausnitzii* labeled with purple lines (**Supplementary Figure 8**). This indicated that the *F. prausnitzii* PFOR sequences were close to each other in the phylogenetic tree (**Supplementary Figure 8**). Using the blastp algorithm, we found that PFOR proteins in seven genera shared a high identity (>80%) and coverage of the PFOR of *F. prausnitzii* A2-165 (**Figures 5A, B**). The PFOR identity of the *Faecalibacterium* genus to the A2-165 strain was over 90%, and the identities of *Gemmiger*, *Roseburia*, *Holdemanella*, *Anaerobutyricum*, *Eubacterium*, and *Agathobacter* were >80% (**Figure 5A**). We then compared the abundances of these PFOR-containing genera in the human cohort. We found that the abundance of *Gemmiger*, *Roseburia*, *Anaerobutyricum*, *Eubacterium*, and *Agathobacter significantly* decreased in UC patients, similar to *Faecalibacterium* (**Figure 5C**). These data suggest that PFOR is conserved and ubiquitous among the gut microbiota, and the abundance of some PFOR-containing genera is also decreased in UC microbiota, indicating that PFOR may mediate microbiota dysbiosis in UC.

## Discussion

In our study, we confirmed that dysbiosis of UC is characterized by disturbed microbial construction with decreased alpha diversity and reduced *F. prausnitzii* abundance. In UC fecal supernatants, IgA levels were significantly increased and reactive to *F. prausnitzii*. In rabbits, *F. prausnitzii* immunization causes intestinal inflammation with increased IgA and altered microbiota. Next, we discovered that PFOR is one of the major antigens of *F. prausnitzii* that is capable of activating PBMCs with elevated cytokine expression. Further scRNA-seq analysis revealed that PFOR mainly affected Tregs and monocytes. In our phylogenetic analysis, PFOR was ubiquitous and conservative among anaerobic bacteria. Both its conservativeness and bioactive function suggest that PFOR is involved in host-microbial crosstalk.

IBD is associated with compositional and metabolic changes in intestinal microbiota (26). It is generally believed that the gut microbiota educates and regulates the immune system, and in turn, intestinal immunity shapes the microbiota (26). The intestinal mucosa secretes IgA and antimicrobial peptides to shape the gut microbiota (27–31). The direct causal relationship between IBD and dysbiosis, especially regarding specific taxonomic changes, remains elusive. Experiments using animal models can help to solve this problem. Generally, there are two types of IBD mouse models: chemically induced and genetically modified. The former involves blunt damage to the mucosa and cannot represent the inciting of IBD. Genetically modified mice often exhibit complete functional loss of a single gene, whereas IBD risk genes are functional in most patients. Neither DSS nor gene knockout was performed in our rabbit experiments. Subcutaneous injection did not directly disturb the intestinal environment, and the rabbit immune system was functional. In the *in vivo* immune experiment, the NMDS test revealed that the fecal microbiome of the QuilA + FP group differed from that of the other groups on the 2nd day. Thus, our results indicated that the microbial change could be secondary to aberrant immunity, although this model did not capture all typical features of UC, especially rabbit weight changes.

A previous study reported that microbial function was more consistently perturbed than composition, with 12% of analyzed pathways changing compared with 2% of genera (32). Proteins may have multiple functions and perform two or more biochemical or biophysical functions (33). Such proteins are called moonlighting proteins and are described further in the online MoonProt Database (34). Many intracellular enzymes are present on the surface of the gut microbiota. They can interact with host cell surface receptors to facilitate adhesion or invasion (35). PFOR is an anaerobic enzyme that facilitates *Trichomonas vaginalis* (*T. vaginalis*) to adhere host cells (36). A polyclonal antibody targeting the 50 kDa recombinant C-terminal region of PFOR reduced the levels of *T. vaginalis* adherence to HeLa cell monolayers in a concentration-dependent manner (37). In our study, purified PFOR was out of the strict anaerobic cytoplasmic environment, and it has been shown to be active in stimulating cytokine expression in PBMCs. Further scRNA-seq analysis confirmed the immunostimulatory effect of PFOR. Furthermore, several PFOR-containing genera have a similar decreasing tendency. These data suggest that PFOR has previously unknown function. However, PFOR may not be the only bioactive protein found in *F. prausnitzii*. Mass Spectrometry of gel bands at 130 and 85 kDa showed several co-existing proteins, such as formate C-acetyltransferase (ATL90706.1), pyruvate, and phosphate dikinase (ATL90002.1). This work provides a new avenue for future host-microbiome interaction studies, and determining the role of other bioactive proteins requires further investigation.

The mechanisms of IgA function in mucosal immunity and intestinal homeostasis have recently been revealed and are still under investigation (38). IgA has been shown to be capable of blocking pathogens from attaching to intestinal epithelial cells by steric hindrance and binding to receptor recognition domains (39, 40). In addition, IgA eliminates antigens and pathogens through a process known as immune exclusion, which includes agglutination, mucus entrapment, and clearance *via* peristalsis (41, 42). Moreover, IgA may have a direct effect on bacterial virulence by suppressing bacterial activity (43). Compared with pathogen-induced IgA, commensal bacteria-induced IgA is generally considered to have low affinity and specificity, which mediates commensal bacterial tolerance and colonization (29). However, IgA coating patterns changed in patients with UC. Elucidating the exact mechanism by which anti-PFOR IgA mediates commensal bacterial deficiency, such as *F. prausnitzii*, requires further exploration.

This study has several shortcomings that need to be pointed out. First, there were no dietary data on UC patients or healthy controls. Whether a possible food-immune-microbiome complex interaction exists is unknown. Intestinal fungi, viruses, protozoa, and mucosal bacteria in UC patients were not evaluated in this study. Further verification using bacterial gene knockout studies is needed to expand our findings. Moreover, only PBMCs from healthy subjects were used in the cell study. What would happen if PFOR encountered PBMCs or intestinal lamina propria mononuclear cells from UC patients, and whether this PFOR stimulated ELISpot assay has any diagnostic value are being investigated in our next study.

In conclusion, our results collectively show that the POFR of *F. prausnitzii* is bioactive for the immune system and mediates host-microbial crosstalk. Conservative microbial proteins, such as PFOR, warrant more attention in future host-microbiome interaction studies.

## Data availability statement

The data presented in the study are deposited in the NCBI repository, accession number PRJNA648093 (human microbiome), PRJNA877845 (rabbit microbiome) and GSE217868 (scRNA-seq).

## Ethics statement

The studies involving human participants were reviewed and approved by Qilu Hospital, Shandong University. The patients/participants provided their written informed consent to participate in this study. The animal study was reviewed and approved by Qilu Hospital, Shandong University.

## Author contributions

K-RW and ML designed this project, created the rabbit models, performed the statistical analysis, and wrote the paper. K-RW, Y-XG, and Y-YL conducted molecular and cellular assays. L-XL. and XG designed the cloning protocol for the PFOR protein. XC recruited human recipients and collected the fecal samples and data. Y-QL supervised the ethical practice of this study. ML conducted the bioinformatics data analysis. All authors contributed to the article and approved the submitted version.

## References

[1] Xavier RJ Podolsky DK. Unravelling the pathogenesis of inflammatory bowel disease. Nature (2007) 448:427–34. doi: 10.1038/nature06005 17653185

[2] Lopez-Siles M Duncan SH Garcia-Gil LJ Martinez-Medina M. Faecalibacterium prausnitzii: from microbiology to diagnostics and prognostics. ISME J (2017) 11:841–52. doi: 10.1038/ismej.2016.176 PMC5364359 28045459

[3] Machiels K Joossens M Sabino J De Preter V Arijs I Eeckhaut V. A decrease of the butyrate-producing species roseburia hominis and faecalibacterium prausnitzii defines dysbiosis in patients with ulcerative colitis. Gut (2014) 63:1275–83. doi: 10.1136/gutjnl-2013-304833 24021287

[4] Varela E Manichanh C Gallart M Torrejón A Borruel N Casellas F. Colonisation by faecalibacterium prausnitzii and maintenance of clinical remission in patients with ulcerative colitis. Aliment. Pharmacol Ther (2013) 38:151–61. doi: 10.1111/apt.12365 23725320

[5] Breyner NM Michon C de Sousa CS Vilas Boas PB Chain F Azevedo VA. Microbial anti-inflammatory molecule (MAM) from faecalibacterium prausnitzii shows a protective effect on DNBS and DSS-induced colitis model in mice through inhibition of NF-κB pathway. Front Microbiol (2017) 8:114. doi: 10.3389/fmicb.2017.00114 28203226 PMC5285381

[6] Zhou L Zhang M Wang Y Dorfman RG Liu H Yu T. Faecalibacterium prausnitzii produces butyrate to maintain Th17/Treg balance and to ameliorate colorectal colitis by inhibiting histone deacetylase 1. Inflammation Bowel Dis (2018) 24:1926–40. doi: 10.1093/ibd/izy182 29796620

[7] Bunker JJ Bendelac A. IgA responses to microbiota. Immunity (2018) 49:211–24. doi: 10.1016/j.immuni.2018.08.011 PMC6107312 30134201

[8] Pabst O. New concepts in the generation and functions of IgA. Nat Rev Immunol (2012) 12:821–32. doi: 10.1038/nri3322 23103985

[9] Montero D Orellana P Gutiérrez D Araya D Salazar JC Prado V. Immunoproteomic analysis to identify shiga toxin-producing escherichia coli outer membrane proteins expressed during human infection. Infection Immun (2014) 82:4767–77. doi: 10.1128/iai.02030-14 PMC4249345 25156722

[10] Gu X Song LJ Li LX Liu T Zhang MM Li Z. Fusobacterium nucleatum causes microbial dysbiosis and exacerbates visceral hypersensitivity in a colonization-independent manner. Front Microbiol (2020) 11:1281. doi: 10.3389/fmicb.2020.01281 32733392 PMC7358639

[11] Kubinak JL Round JL. Do antibodies select a healthy microbiota? Nat Rev Immunol (2016) 16:767–74. doi: 10.1038/nri.2016.114 PMC9004535 27818504

[12] Palm NW de Zoete MR Cullen TW Barry NA Stefanowski J Hao L. Immunoglobulin a coating identifies colitogenic bacteria in inflammatory bowel disease. Cell (2014) 158:1000–10. doi: 10.1016/j.cell.2014.08.006 PMC4174347 25171403

[13] Lin R Chen H Shu W Sun M Fang L Shi Y. Clinical significance of soluble immunoglobulins a and G and their coated bacteria in feces of patients with inflammatory bowel disease. J Trans Med (2018) 16:359. doi: 10.1186/s12967-018-1723-0 PMC6296095 30558634

[14] Stoll ML Kumar R Morrow CD Lefkowitz EJ Cui X Genin A. Altered microbiota associated with abnormal humoral immune responses to commensal organisms in enthesitis-related arthritis. Arthritis Res Ther (2014) 16:486. doi: 10.1186/s13075-014-0486-0 25434931 PMC4272554

[15] Asshauer KP Wemheuer B Daniel R Meinicke P. Tax4Fun: predicting functional profiles from metagenomic 16S rRNA data. Bioinformatics (2015) 31:2882–4. doi: 10.1093/bioinformatics/btv287 PMC4547618 25957349

[16] Leonardi I Nicholls F Atrott K Cee A Tewes B Greinwald R. Oral administration of dextran sodium sulphate induces a caecum-localized colitis in rabbits. Int J Exp Pathol (2015) 96:151–62. doi: 10.1111/iep.12117 PMC4545426 25716348

[17] Ni X Xu K Zhao Y Li J Wang L Yu F. Single-cell analysis reveals the purification and maturation effects of glucose starvation in hiPSC-CMs. Biochem Biophys Res Commun (2021) 534:367–73. doi: 10.1016/j.bbrc.2020.11.076 33279112

[18] Yu GC Smith DK Zhu HC Guan Y Lam TTY. GGTREE: an r package for visualization and annotation of phylogenetic trees with their covariates and other associated data. Methods Ecol Evol (2017) 8:28–36. doi: 10.1111/2041-210x.12628

[19] Jin DX Zou HW Liu SQ Wang LZ Xue B Wu. The underlying microbial mechanism of epizootic rabbit enteropathy triggered by a low fiber diet. Sci Rep (2018) 8:12489. doi: 10.1038/s41598-018-30178-2 30131509 PMC6104036

[20] Petrovsky N Aguilar JC. Vaccine adjuvants: current state and future trends. Immunol Cell Biol (2004) 82:488–96. doi: 10.1111/j.0818-9641.2004.01272.x 15479434

[21] Chassaing B Aitken JD Malleshappa M Vijay-Kumar M. Dextran sulfate sodium (DSS)-induced colitis in mice. Curr Protoc Immunol (2014) 104:15 25 11–15 25 14. doi: 10.1002/0471142735.im1525s104 PMC3980572 24510619

[22] Seo JH Youn JH Kim EA Jun JS Park JS Yeom JS. Helicobacter pylori antigens inducing early immune response in infants. J Korean Med Sci (2017) 32:1139–46. doi: 10.3346/jkms.2017.32.7.1139 PMC5461318 28581271

[23] Maynard CL Harrington LE Janowski KM Oliver JR Zindl CL Rudensky AY. Regulatory T cells expressing interleukin 10 develop from Foxp3+ and Foxp3- precursor cells in the absence of interleukin 10. Nat Immunol (2007) 8:931–41. doi: 10.1038/ni1504 17694059

[24] Cong Y Feng T Fujihashi K Schoeb TR Elson CO. A dominant, coordinated T regulatory cell-IgA response to the intestinal microbiota. Proc Natl Acad Sci U.S.A. (2009) 106:19256–61. doi: 10.1073/pnas.0812681106 PMC2780781 19889972

[25] Feng T Elson CO Cong Y. Treg cell-IgA axis in maintenance of host immune homeostasis with microbiota. Int Immunopharmacol (2011) 11:589–92. doi: 10.1016/j.intimp.2010.11.016 PMC3078992 21111079

[26] Ni J Wu GD Albenberg L Tomov VT. Gut microbiota and IBD: causation or correlation? Nat Rev Gastroenterol Hepatol (2017) 14:573–84. doi: 10.1038/nrgastro.2017.88 PMC5880536 28743984

[27] Bollinger RR Everett ML Palestrant D Love SD Lin SS Parker W. Human secretory immunoglobulin a may contribute to biofilm formation in the gut. Immunology (2003) 109:580–7. doi: 10.1046/j.1365-2567.2003.01700.x PMC1782994 12871226

[28] Bollinger RR Everett ML Wahl SD Lee YH Orndorff PE Parker W. Secretory IgA and mucin-mediated biofilm formation by environmental strains of escherichia coli: Role of type 1 pili. Mol Immunol (2006) 43:378–87. doi: 10.1016/j.molimm.2005.02.013 16310051

[29] Donaldson GP Ladinsky MS Yu KB Sanders JG Yoo BB Chou WC. Gut microbiota utilize immunoglobulin a for mucosal colonization. Science (2018) 360:795–800. doi: 10.1126/science.aaq0926 29724905 PMC5973787

[30] Lee SM Donaldson GP Mikulski Z Boyajian S Ley K Mazmanian SK. Bacterial colonization factors control specificity and stability of the gut microbiota. Nature (2013) 501:426–9. doi: 10.1038/nature12447 PMC3893107 23955152

[31] Peterson DA McNulty NP Guruge JL Gordon JI. IgA response to symbiotic bacteria as a mediator of gut homeostasis. Cell Host Microbe (2007) 2:328–39. doi: 10.1016/j.chom.2007.09.013 18005754

[32] Morgan XC Tickle TL Sokol H Gevers D Devaney KL Ward DV. Dysfunction of the intestinal microbiome in inflammatory bowel disease and treatment. Genome Biol (2012) 13:R79. doi: 10.1186/gb-2012-13-9-r79 23013615 PMC3506950

[33] Jeffery CJ. Moonlighting proteins. Trends Biochem Sci (1999) 24:8–11. doi: 10.1016/s0968-0004(98)01335-8 10087914

[34] Mani M Chen C Amblee V Liu H Mathur T Zwicke G. MoonProt: a database for proteins that are known to moonlight. Nucleic Acids Res (2015) 43:D277–282. doi: 10.1093/nar/gku954 PMC4384022 25324305

[35] Jeffery CJ. Intracellular/surface moonlighting proteins that aid in the attachment of gut microbiota to the host. AIMS Microbiol (2019) 5:77–86. doi: 10.3934/microbiol.2019.1.77 31384704 PMC6646928

[36] Song HO. Influence of 120 kDa Pyruvate: Ferredoxin oxidoreductase on pathogenicity of trichomonas vaginalis. Korean J Parasitol (2016) 54:71–4. doi: 10.3347/kjp.2016.54.1.71 PMC4792314 26951982

[37] Meza-Cervantez P González-Robles A Cárdenas-Guerra RE Ortega-López J Saavedra E Pineda E. Pyruvate:ferredoxin oxidoreductase (PFO) is a surface-associated cell-binding protein in trichomonas vaginalis and is involved in trichomonal adherence to host cells. Microbiol (Reading England) (2011) 157:3469–82. doi: 10.1099/mic.0.053033-0 22130740

[38] Mantis NJ Rol N Corthesy B. Secretory IgA's complex roles in immunity and mucosal homeostasis in the gut. Mucosal Immunol (2011) 4:603–11. doi: 10.1038/mi.2011.41 PMC3774538 21975936

[39] Helander A Miller CL Myers KS Neutra MR Nibert ML. Protective immunoglobulin a and G antibodies bind to overlapping intersubunit epitopes in the head domain of type 1 reovirus adhesin sigma1. J Virol (2004) 78:10695–705. doi: 10.1128/JVI.78.19.10695-10705.2004 PMC516417 15367636

[40] Helander A Silvey KJ Mantis NJ Hutchings AB Chandran K Lucas WT. The viral sigma1 protein and glycoconjugates containing alpha2-3-linked sialic acid are involved in type 1 reovirus adherence to m cell apical surfaces. J Virol (2003) 77:7964–77. doi: 10.1128/jvi.77.14.7964-7977.2003 PMC161912 12829836

[41] Mantis NJ Forbes SJ. Secretory IgA: Arresting microbial pathogens at epithelial borders. Immunol Invest. (2010) 39:383–406. doi: 10.3109/08820131003622635 20450284 PMC3774547

[42] Phalipon A Cardona A Kraehenbuhl JP Edelman L Sansonetti PJ Corthesy B. Secretory component: A new role in secretory IgA-mediated immune exclusion in vivo. Immunity (2002) 17:107–15. doi: 10.1016/s1074-7613(02)00341-2 12150896

[43] Forbes SJ Bumpus T McCarthy EA Corthesy B Mantis NJ. Transient suppression of shigella flexneri type 3 secretion by a protective O-antigen-specific monoclonal IgA. mBio (2011) 2:e00042–00011. doi: 10.1128/mBio.00042-11 PMC3101778 21610121

