# Supplementary material for: Pyruvate: Ferredoxin oxidoreductase is involved in IgA-related microbiota dysbiosis and intestinal inflammation
Source: Front Immunol. 2022 Dec 7;13:1040774. doi: 10.3389/fimmu.2022.1040774 (PMC9782971; doi:10.3389/fimmu.2022.1040774)
Supplement: Supplementary file 4 [file DataSheet_4.docx]

**Supplementary Material and Methods**

**Pyruvate: ferredoxin oxidoreductase is involved in IgA-related microbiota dysbiosis and intestinal inflammation**

Kairuo Wang^1,2^, Yixuan Guo^1,3,4^, Yuanyuan Liu^1,3,4^, Xiao Cui^1,3,4^, Xiang Gu^1,3,4^, Lixiang Li^1,3,4^, Yanqing Li^1,3,4^, Ming Li^1,3,4,5^

1. Department of Gastroenterology, Qilu Hospital, Shandong University, Jinan, Shandong, China

2. Department of Gastroenterology, Shanghai Tenth People's Hospital, School of Medicine, Tongji University, Shanghai, China.

3. Laboratory of Translational Gastroenterology, Qilu Hospital, Shandong University, Jinan, Shandong, China

4. Robot Engineering Laboratory for Precise Diagnosis and Therapy of Gastrointestinal Tumor, Qilu Hospital, Shandong University, Jinan, Shandong, China

5. Shandong Provincial Clinical Research Center for digestive disease, Qilu Hospital, Shandong University, Jinan, Shandong, China

Correspondence: Ming Li

Email: limingecho@126.com


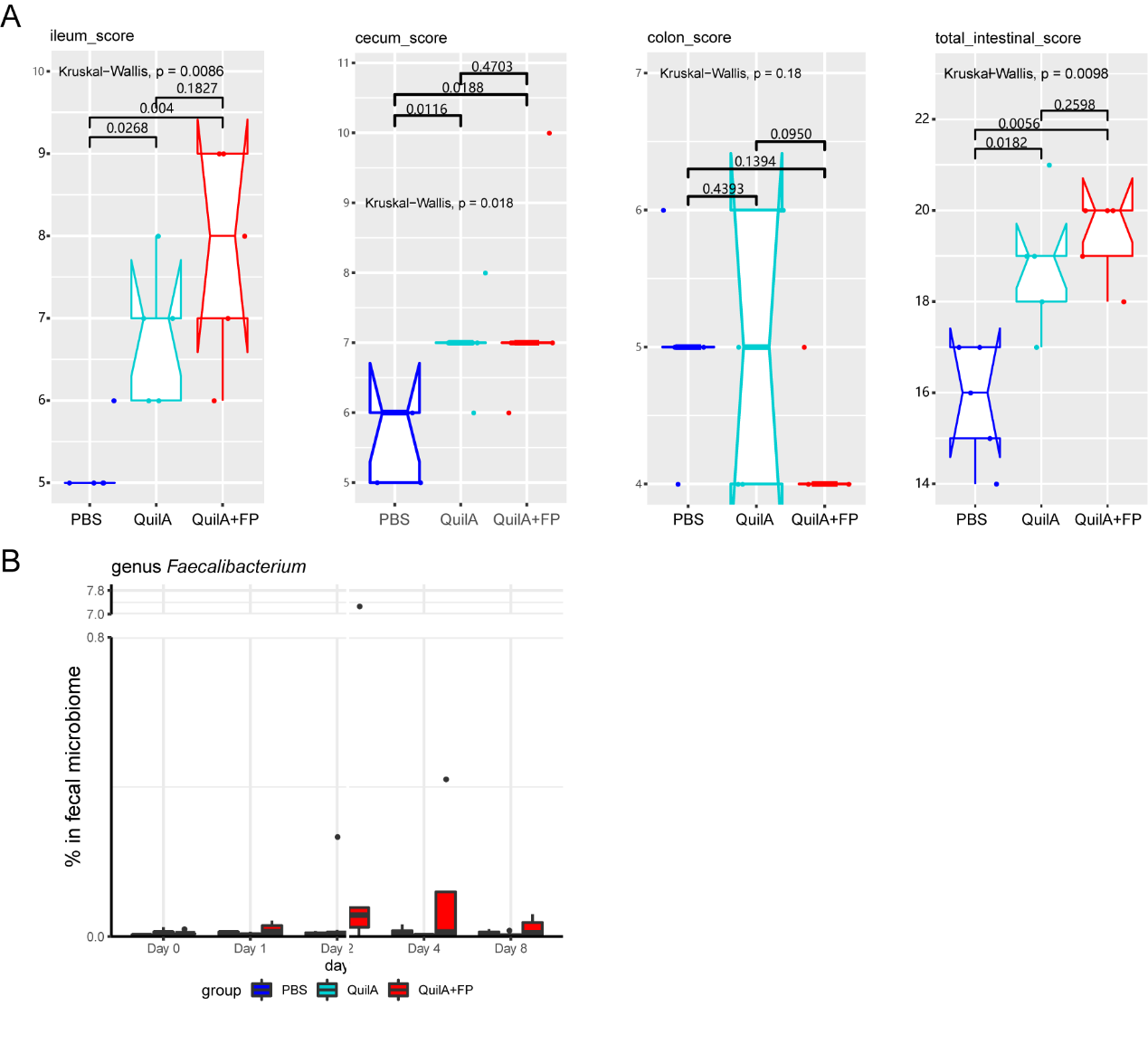


**Supplementary Figure 1** (A) The pathological score of *F. prausnitzii* immunization on rabbits. (B) *Faecalibacterium* genus abundance of *F. prausnitzii* immunization on rabbits.


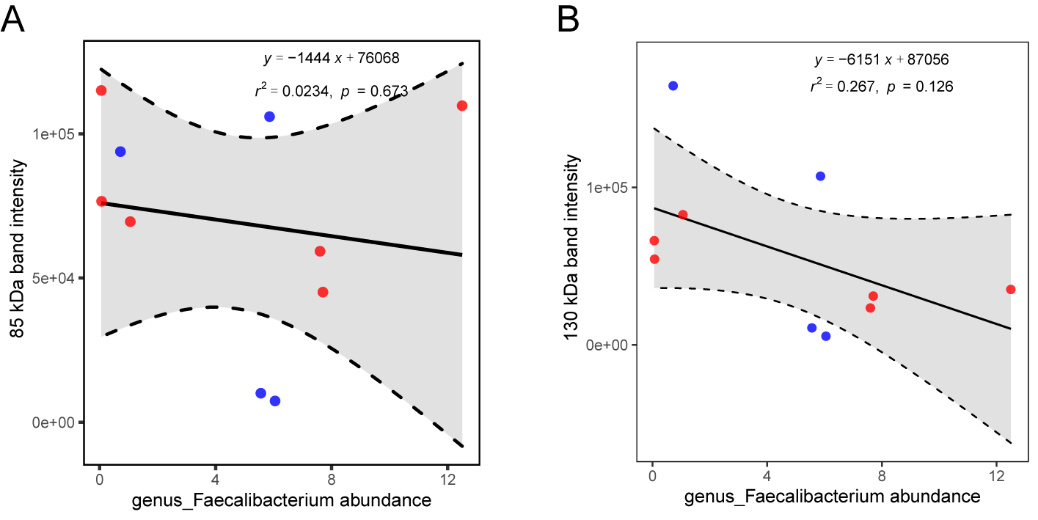


**Supplementary Figure 2** Spearman correlations between *Faecalibacterium* abundance and 85 kDa band intensity (A) and 130 kDa band intensity (B).


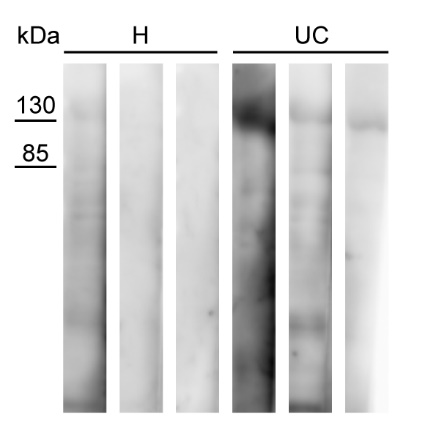


**Supplementary Figure 3** Representative images of western blot using PFOR protein as substrates, UC or healthy fecal supernatants as primary antibody, and anti-IgA antibody as secondary antibody.


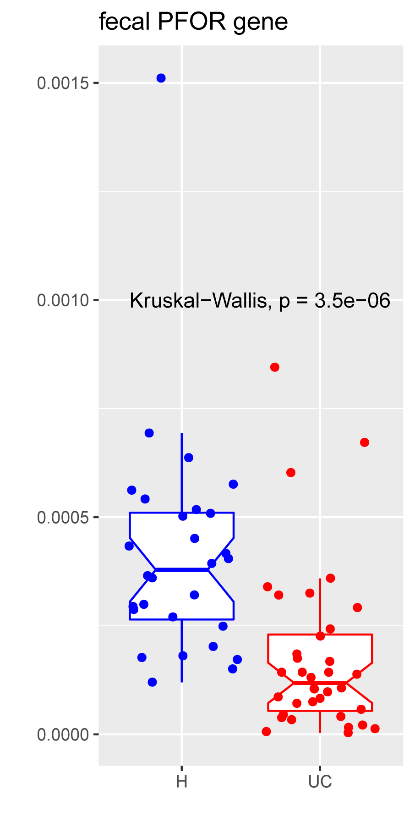


**Supplementary Figure 4** The PFOR gene abundance of healthy controls and UC patients predicted by Tax4Fun.


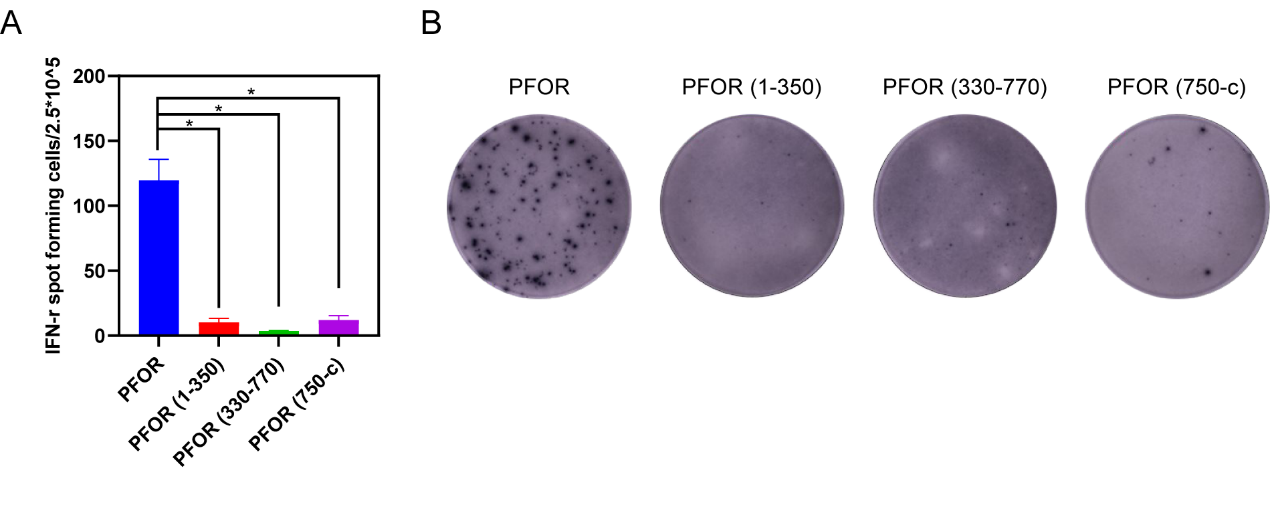


**Supplementary Figure 5** The effect of fractions of PFOR on PBMCs. (A) Analysis of ELISpot detecting IFN-γ expression in PBMC. (B) Spot figures of ELISpot detecting IFN-γ expression in PBMC.


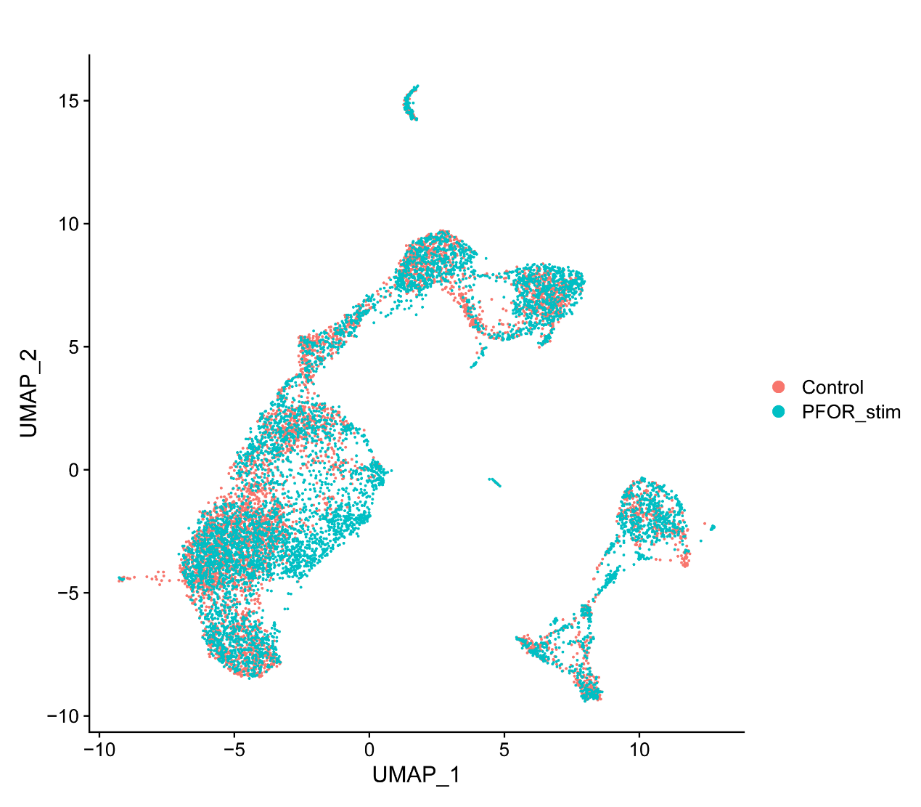


**Supplementary Figure 6** UMAP reduction plot of the PBMC incubated with PFOR (light blue) and control (light red). The two sample overlaps well, indicating little or no batch effect after integration.


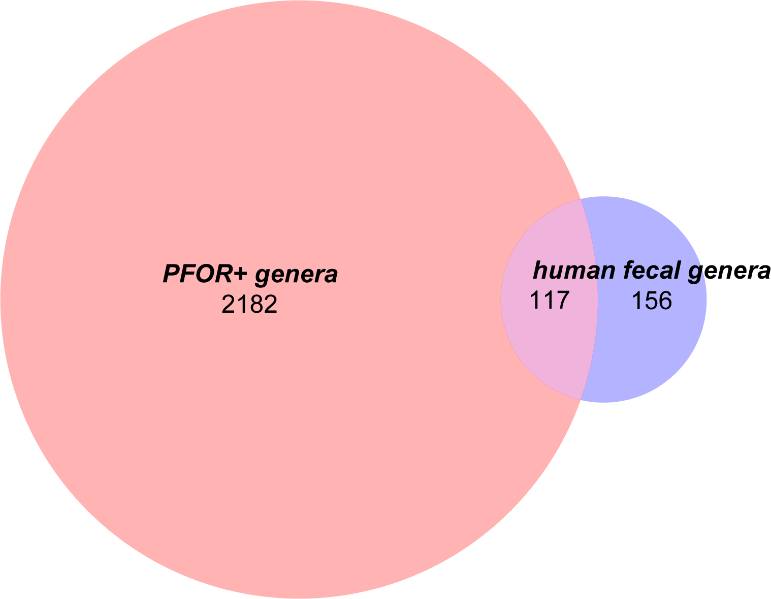


**Supplementary Figure 7** Veen diagram of PFOR^+^ genera and human fecal genera.


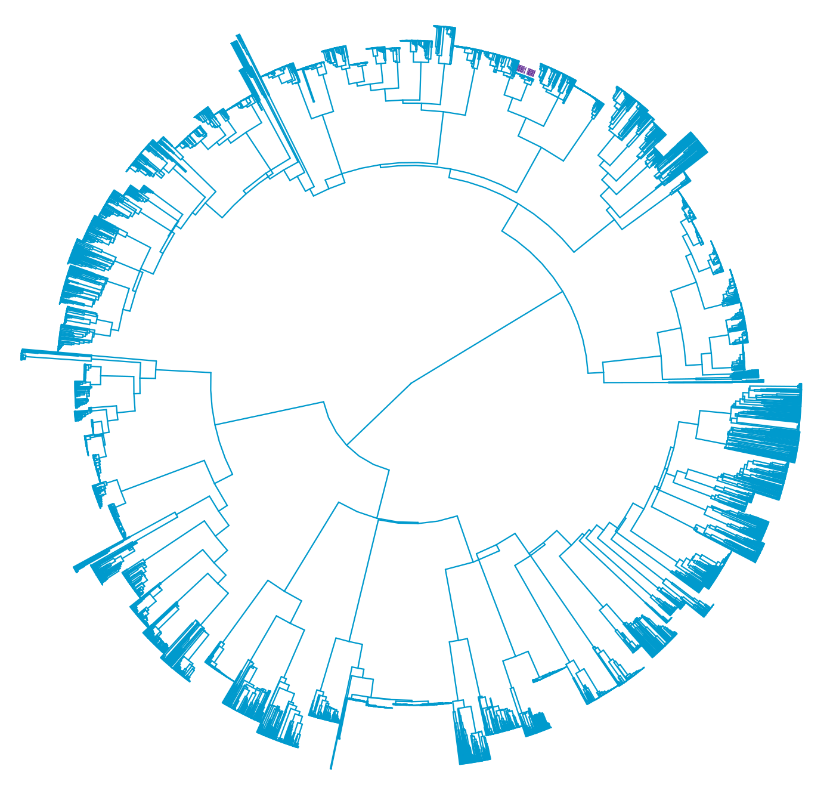


**Supplementary Figure 8** The phylogenetic tree of all PFOR protein of human fecal microbiome. The purple lines indicated the PFOR sequences of *F. prausnitzii.*

**Supplementary Table 1. Clinical characteristics of healthy controls and ulcerative colitis patients, related to Figure 1.**

|  | Healthy controls (n=28) | Ulcerative colitis (n=36) | | *P*-value |
| --- | --- | --- | --- | --- |
| Age (mean years ± SD) | 46.39 ± 10.41 | | 50.81 ± 14.37 | 0.176 |
| Male (n, %) | 15, 40.5% | | 22, 59.5% | 0.367 |
| Body mass index (kg/m2) | 24.85 ± 3.84 | | 22.61 ± 3.18 | 0.014 |
| Smokers (n, %) |  | | 12, 18.8% |  |
| Disease behavior |  | |  |  |
| Rectum (E1) |  | | 7, 19.4% |  |
| Sigmoid + left colon (E2) |  | | 16, 44.4% |  |
| Entire colon (E3) |  | | 13, 36.1% |  |
| Medication |  | |  |  |
| Mesalazine |  | | 24, 66.6% |  |
| Steroids |  | | 14, 38.9% |  |
| Azathioprine |  | | 0, 0% |  |
| Sulfasalazine |  | | 4, 11.1% |  |
| Anti-TNF-α |  | | 2, 5.5% |  |
| Inflammation markers |  | |  |  |
| CRP levels (mg/L) |  | | 14.14 ± 32.49 |  |
| Sedimentation rate (mm/h) |  | | 17.59 ± 22.25 |  |
| PCT (ng/ml) |  | | 0.024 ± 0.066 |  |
| Albumin (g/L) |  | | 35.04 ± 12.16 |  |

**Supplementary Table 2. Scoring system of histological changes, related to Figure 2.**

|  | **Morphological features** | | | **Inflammation** | |
| --- | --- | --- | --- | --- | --- |
|  | **Villous stunting** | **Villous epithelial injury** | **Crypt distortion** | **Intraepithelial lymphocytes (IEL)** | [**Lamina propria**](http://www.baidu.com/link?url=oNOgg_oKBtplgxhQNcKU_sw3gbngLRoqXrAiG1F-M8BIP4TOcUl_JSuH4d9vmE-IcXB4RLDnEYsYoCYYFVYuTENFveaCj7ogtnFM2_nNZzR1XSqXGF6Uxxhc7X9UaJyS) **lymphocytes and plasma cells** |
| 1 | Normal mucosa | Normal mucosa | Normal mucosa | 5-10 IEL/50 epithelial cells | 25% of the villous lamina propria |
| 2 | Mild villous stunting | Mild villous epithelial injury | Mild crypt distension, hyperplasia and distortion | 11-30 IEL/50 epithelial cells | 25-50% of the villous lamina propria |
| 3 | Moderate villous stunting | Moderate villous epithelial injury | Moderate crypt distension, hyperplasia and distortion | 31-50 IEL/50 epithelial cells, may be focally clustered | 50-75% of the villous lamina propria |
| 4 | Marked villous stunting | Marked villous epithelial injury | Marked crypt distension, hyperplasia and distortion | 51-100 IEL/50 epithelial cells, may be clustered and at all levels of the epithelium | 75-100% of the villous lamina propria |

**Supplementary Table 3. Oligonucleotide sequences, related to Figure 2 and Figure 3.**

| Primer Target | Sequencce（5’-3’） | Length（bp） |
| --- | --- | --- |
| rabbit IFN-γ | F: TCGGTGGATGATCGGCTGGTC  R: CGACCTCGAAACAGCGTCTGAC | 122 |
| rabbit IL-22 | F: AGGAGGCCAATTCGGCAGAT  R: ATCAGACTCGGGGAACAGCA | 146 |
| rabbit IL-6 | F: GCTGAAGACGACCACGATCCAC  R: CAAGCAAGGACACCCGCACTC | 103 |
| rabbit IL-1β | F: GTCTTCCTAAAGCAAGCCTTAC  R: GGGGTGTCACAATCTGTTTC | 92 |
| rabbit COX-2 | F: AGCTCACTCACACAAGCACA  R: AGGATTCGTAGGGAGGGCAG | 106 |
| rabbit GAPDH | F: AAGGTCGGAGTGAACGGAT  R: TCGCTCCTGGAAGATGGT | 228 |
| human IFN-γ | F: GAGATGACTTCGAAAAGCTGAC  R: CCTTTTTCGCTTCCCTGTTTTA | 128 |
| human IL-1β | F: AGCTACGAATCTCCGACCAC  R: CGTTATCCCATGTGTCGAAGAA | 186 |
| human IL-6 | F: ACTCACCTCTTCAGAACGAATTG  R: CCATCTTTGGAAGGTTCAGGTTG | 149 |
| human IL-17a | F: TCAACCCGATTGTCCACCAT  R: GAGTTTAGTCCGAAATGAGGCTG | 136 |
| human GAPDH | F: GTGGACCTGACCTGCCGTCTAG  R: GAGTGGGTGTCGCTGTTGAAGTC | 149 |

**Supplementary Table 4. Proteins identified by LC-MS/MS and bioinformatics, related to Figure 3.**

| **Band No.** | **Hits** | **Protein Mass^a^** | **No. of Peptide^b^** | **Sequence Header^c^** | **Relative Abundance** | **Probability** | **No. of Unique Peptide** |
| --- | --- | --- | --- | --- | --- | --- | --- |
|  | 1 | 129607.11 | 260 | ATL90210.1 pyruvate:ferredoxin (flavodoxin) oxidoreductase | 54.4% | 99.0% | 49 |
|  | 2 | 84939.88 | 58 | ATL90706.1 formate C-acetyltransferase | 12.0% | 99.0% | 26 |
|  | 3 | 133462.56 | 51 | RGC40351.1 DNA-directed RNA polymerase subunit beta | 7.1% | 99.0% | 38 |
|  | 1 | 84939.88 | 264 | ATL90706.1 formate C-acetyltransferase | 31.1% | 99.0% | 39 |
|  | 2 | 84815.88 | 212 | AXA82511.1 formate C-acetyltransferase | 25.2% | 99.0% | 29 |
|  | 3 | 76012.4 | 101 | WP_005932243.1 twin-arginine translocation signal domain-containing protein | 6.3% | 99.0% | 19 |
|  | …… |  |  |  |  |  |  |
|  | 11 | 129607.11 | 25 | ATL90210.1 pyruvate:ferredoxin (flavodoxin) oxidoreductase | 0.5% | 99.0% | 14 |

a) Protein Mass: The calculated molecular weight of each identified protein based on its amino acid sequence present in the current NR database. b) No. of Peptide: The number of peptides sequenced by LC-MS/MS in each identified protein. c) Sequencing Header: The header of an identified protein present in NR database.

**Supplementary Data 1** The genus level comparison between UC patients and the healthy.

**Supplementary Data 2** MALDI-TOF/TOF results of isolated gel bands.

**Supplementary Data 3** Human PFOR entry from Uniport.
